# Research on autonomous navigation system of greenhouse electric crawler tractor based on LiDAR

**DOI:** 10.3389/fpls.2024.1377269

**Published:** 2024-05-15

**Authors:** Huiping Guo, Yi Li, Hao Wang, Tingwei Wang, Linrui Rong, Haoyu Wang, Zihao Wang, Chensi Wang, Jiao Zhang, Yaobin Huo, Shaomeng Guo

**Affiliations:** ^1^College of Mechanical and Electronic Engineering, Northwest Agriculture and Forestry University, Yangling, Shaanxi, China; ^2^Northern Agricultural Equipment Scientific Observation and Experimental Station, Ministry of Agriculture and Rural Affairs, Yangling, Shaanxi, China; ^3^Project Control Department, BYD Automobile Company Limited, Xi’an, China; ^4^Xi ’an Dongyang Machinery Company Limited, Xi’an, China

**Keywords:** electric crawler tractor, greenhouse, lidar, autonomous navigation, path planning

## Abstract

The application of autonomous navigation technology of electric crawler tractors is an important link in the development of intelligent greenhouses. Aiming at the characteristics of enclosed and narrow space and uneven ground potholes in greenhouse planting, to improve the intelligence level of greenhouse electric crawler tractors, this paper develops a navigation system of electric crawler tractors for the greenhouse planting environment based on LiDAR technology. The navigation hardware system consists of five modules: the information perception module, the control module, the communication module, the motion module, and the power module. The software system is composed of three layers: the application layer, the data processing layer, and the execution layer. The developed navigation system uses LiDAR, Inertial Measurement Unit (IMU) and wheel speed sensor to sense the greenhouse environment and the crawler tractor’s information, employs the Gmapping algorithm to build the greenhouse environment map, and utilizes the adaptive Monte Carlo positioning algorithm for positioning. The simulation test of different global path planning algorithms in Matlab shows that the A* algorithm obtains the optimal overall global path. In the scene of map 5, the path planned by the A* algorithm is the most significant, and the number of inflection points is reduced by 40.00% and 87.50%, respectively; meanwhile, the path length is the same as that of the Dijkstra algorithm, but the runtime is reduced by 68.87% and 81.49%, respectively; compared with the RRT algorithm, the path length is reduced by 7.27%. Therefore, the A* algorithm and the Dynamic Window Approach (DWA) method are used for tractor navigation and obstacle avoidance, which ensures global path optimality while also achieving effective local path planning for obstacle avoidance. The test results suggest that the maximum lateral deviation of the built map is 6 cm, and the maximum longitudinal deviation is 16 cm, which meets the requirement of map accuracy. Additionally, the results of the navigation accuracy test indicate that the maximum lateral deviation of navigation is less than 13 cm, the average lateral deviation is less than 7 cm, and the standard lateral deviation is less than 8 cm. The maximum heading deviation is less than 14°, the average heading deviation is less than 7°, and the standard deviation is less than 8°. These results show that the developed navigation system meets the navigation accuracy requirements of electric crawler tractors in the greenhouse environment.

## 1 Introduction

By the end of 2021, China boasted over 28 million facility greenhouses, covering an area surpassing 40 million mu, making it the largest in terms of land area in the world (Hu et al., 2024). The mechanization of greenhouse cultivation has also become a hot research topic. Due to the enclosed and narrow space in greenhouses, traditional tractors, which cause severe pollution, are being replaced, thus research on electric crawler tractor has become one of the important directions in the study of greenhouse machinery. They serve as traction machinery to complete tasks such as plowing, rotary tillage, seeding, and harvesting within the greenhouse.

Given the enclosed environment of greenhouses, which are characterized by high temperatures and humidity, and the harsh working conditions, the automation of electric crawler tractor is particularly important due to the increased intensity of manual labor (Liu et al., 2022). The key to achieving automation in electric crawler tractor is autonomous navigation technology (Mahmud et al., 2019). Autonomous navigation mainly includes mapping, positioning, and path planning, and the accurate perception of environmental information is critical for precise navigation. Currently, the sensors used for environmental perception mainly include Global Navigation Satellite System (GNSS), vision, LiDAR, attitude sensor, and other sensors (İrsel and Altinbalik, 2018; Jiang et al., 2023; Jia et al., 2015; Arad et al., 2020; Zhang et al., 2020).

Autonomous navigation technology in industrial environments is relatively mature, often employing multi-sensor fusion for navigation, which provides high accuracy but involves higher costs, making it not entirely suitable for the agricultural production environment (Fu et al., 2023a, Fu et al., 2023b). Currently, in agricultural environments, GNSS technology is mainly used for autonomous navigation in fields or orchards (Wang et al., 2014). Zhang et al. (2013) developed a GNSS-based autonomous navigation system for combine harvesters, used for harvesting wheat and rice. Compared to field operations, orchards present challenges like canopy cover, which can affect GNSS signals, making it difficult to ensure high-precision work. Many scholars have introduced IMU, wheel speed sensor, vision, and other sensors on top of GNSS positioning to improve the positioning accuracy of equipment. Liu et al. (2018) designed a navigation operation system for a high clearance sprayer based on GNSS and inertial sensors, meeting the spraying operation requirements in three environments: cement pavements, dry fields, and paddy fields, with good stability and control precision. Kurashiki et al. (2010) used GPS and laser range finder to achieve autonomous positioning of autonomous vehicles in orchards, and the experimental results indicated that the average error in the lateral direction was about 20 cm, meeting the accuracy requirements for orchard navigation. Though the fusion of GNSS with other sensors can solve the problem of navigation accuracy under branch coverage to a good extent, in completely enclosed greenhouse environments, GNSS signals are significantly obstructed, making it difficult to meet the requirements for precise positioning.

Vision sensors can capture a wealth of information in enclosed environments and have been widely used in agricultural navigation applications in recent years (Tan et al., 2020). Wang et al. (2013) utilized monocular vision for tractor navigation, significantly reducing the operator’s labor intensity and achieving autonomous navigation of agricultural tractors. Radcliffe et al. (2018) designed a navigation platform based on vision and ultrasonic sensors, using a multi-spectral camera to capture images of orchards and integrating them with background information of the canopy and sky. Machine vision was employed to extract the road in the center of tree rows, using ultrasound to measure distances. Although visual sensors can solve navigation problems in enclosed environments to a certain extent, they are susceptible to factors such as lighting and shadows in the greenhouse environment, making it difficult to achieve stable operation around the clock.

Laser sensors are widely used in enclosed environments such as indoors, offering many advantages including high ranging accuracy, good resolution, strong anti-interference capability, small size, and light weight. Many scholars have used LiDAR to solve the environmental perception and positioning issues of material handling and inspection robots, providing support for autonomous navigation (Jia et al., 2015; Jiang et al., 2022). Single LiDAR can enable navigation on smooth road surfaces, but motion-induced point cloud distortion in environmental perception can lead to inaccuracies in positioning and mapping, causing information biases. LiDAR integration with pose sensors can enhance the precision of positioning and mapping of LiDAR-based navigation to some extent by rectifying these distortions. However, on navigation of tractors driving on uneven terrains, LiDAR solely integrating with IMU struggles to address the accumulation of IMU measurement biases. Moreover, LiDAR solely integrating with wheel speed sensor fails to adequately address the significant errors in pose estimation caused by signal drift. These indicate that fusing LiDAR with a single type of pose sensor is insufficient to resolve the substantial positioning and mapping errors induced by uneven surfaces. Therefore, the integration of LiDAR with multiple pose sensors is crucial for achieving precise positioning and mapping on uneven terrains.

Although a single LiDAR can solve indoor navigation problems to a certain extent, in greenhouse environments, the ground surfaces on which tractors work are rough and uneven dirt roads. In the process of environmental perception, a single LiDAR encounters point cloud distortion and is affected by the unevenness of the ground, leading to increased cumulative errors in positioning and mapping, deviations in environmental perception, and affecting the accuracy and completeness of mapping. The fusion of LiDAR and attitude sensors can obtain feature information of the greenhouse environment, information about obstacles around the tractor, and the state information of the tractor itself, enabling more accurate and reliable autonomous navigation.

**Figure 1:**
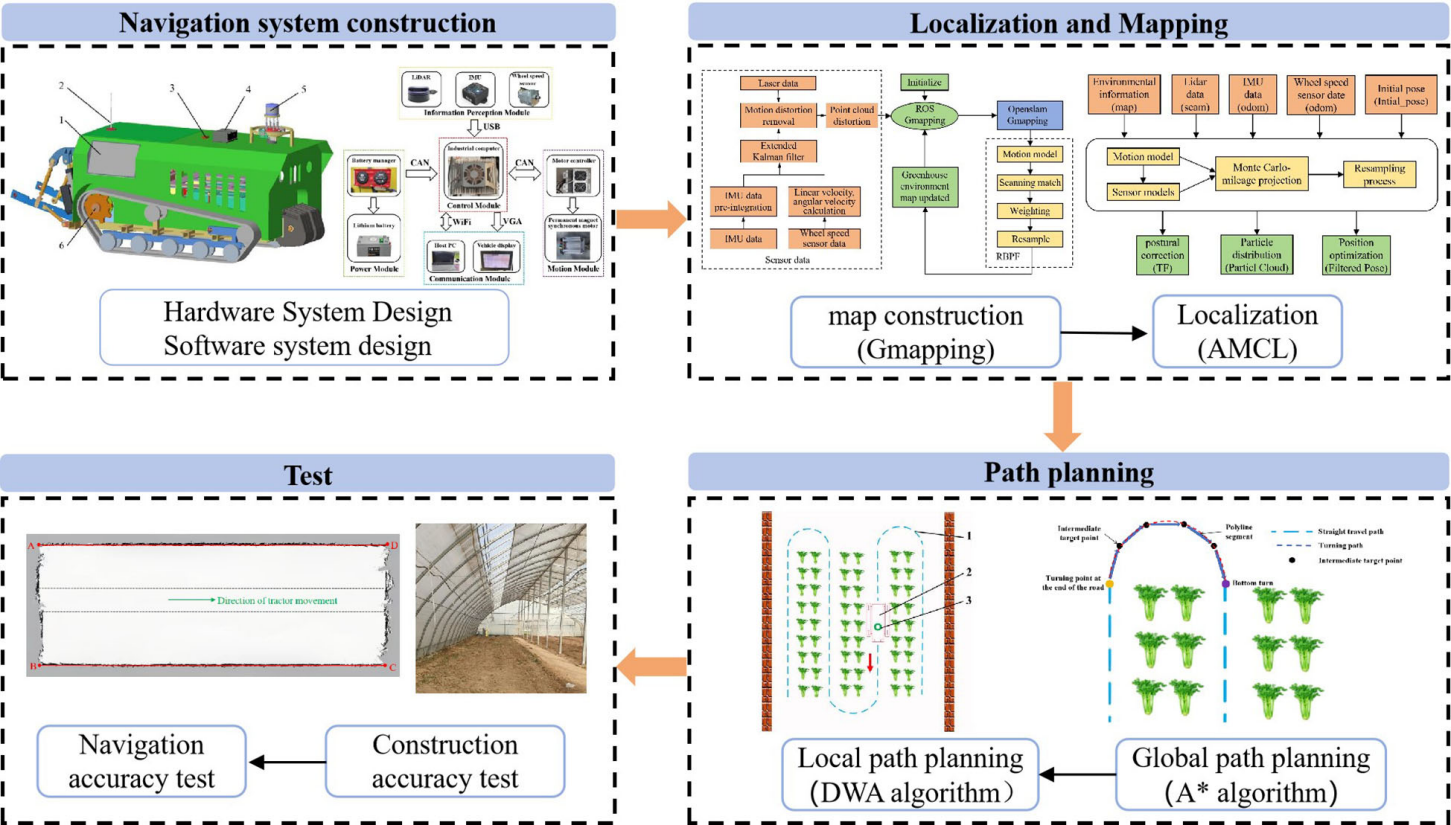
Overall structure diagram.

In response to these challenges, this study, based on a remotely operated electric crawler tractor for greenhouses developed by our team, has designed an autonomous navigation system suitable for operating on uneven terrains of greenhouses. This system acquires the environmental data from LiDAR, and the pose information from IMU and wheel speed sensor of the tractor. The system employs an Extended Kalman Filter algorithm to fuse the data from IMU and wheel speed sensor, correcting motion-induced distortions in LiDAR point clouds, thereby improving the accuracy of positioning, mapping, and navigation on uneven surfaces of greenhouses. The overall structure of the research is shown in **Figure 1**. The main contributions are summarized as follows:

(1) Tailored to the characteristics of greenhouse operation environments, an autonomous navigation system for the tractor was built on the developed remote-controlled greenhouse electric crawler tractor, utilizing LiDAR, IMU, and wheel speed sensor, encompassing the design of both hardware and software systems;
(2) It proposes a mapping method for the greenhouse environment using the Gmapping algorithm, employs the Adaptive Monte Carlo Localization algorithm for positioning, and uses the A* algorithm for global path planning and DWA for obstacle avoidance, validated through simulations and comparison with operational needs;
(3) At the software layer of the electric tractor, the navigation system development is based on the Robot Operating System (ROS), with the mapping performance and navigation accuracy of the navigation system validated within a greenhouse.

The remainder of this paper is organized as follows: The second section discusses the design of the electric crawler tractor navigation system, including mapping, positioning, and path planning of the navigation system; the third section addresses the testing experiments for mapping, positioning, and navigation accuracy of the designed navigation system, along with an analysis of the test results; finally, the conclusion.

## 2 Related works

LiDAR is not affected by lighting conditions, possesses strong anti-interference capability, and can accurately measure distances to surrounding objects. It has been widely applied in autonomous navigation for devices in various enclosed spaces (Hiremath et al., 2014). Li et al. (2023a) used LiDAR to construct maps of unknown environments to achieve autonomous path planning and navigation for mobile robots indoors. Li et al. (2023b) utilized LiDAR to acquire information on complex, intelligent factory environments to enhance the precise positioning and navigation performance of AGV (Automated Guided Vehicles) in an intelligent factory setting. While a single LiDAR can achieve indoor navigation, due to point cloud distortion during the autonomous navigation process, cumulative errors can occur over time, affecting navigation precision and failing to obtain accurate positioning.

Many scholars have attempted to solve navigation precision issues by integrating different sensors with LiDAR. Hou et al. (2020) developed a navigation system for transport robots based on dual LiDAR, matching the data from encoders with LiDAR point cloud data to obtain the environmental map and the robot’s pose information, thereby increasing the robot’s scanning range and map-building efficiency. Hu et al. (2023) adopted LiDAR and laser receivers to design a robot positioning system based on laser sensing, by acquiring the point cloud on the robot’s laser receiver through the laser emitted from the LiDAR during scanning. Simultaneously, the laser receiver senses the scanning laser, integrating the time difference of sensing scanning laser and the laser receiver’s point cloud features to localize the agricultural robot. Dang et al. (2021) leveraged the complementary characteristics of millimeter-wave radar and LiDAR, proposing an efficient and precise method of detecting, recognizing, and eliminating moving objects through sensor fusion and data association to enhance the performance of positioning and mapping. The above research utilizes external sensors to perceive environmental information for positioning, thus achieving navigation solutions, but their costs are relatively high.

Pose sensors such as IMU and wheel speed sensor are relatively cost-effective, not easily disturbed by external environments, and can provide information on the robot’s own pose. Scholars have proposed the fusion of LiDAR with IMU and wheel speed sensor, which can effectively compensate for the limitations of using LiDAR alone, enhancing the robustness and accuracy of positioning and mapping. The IMU measures the acceleration of the robot’s motion via an accelerometer and measures the angular velocity of motion via a gyroscope, calculating the current attitude through an attitude fusion algorithm (Pu et al., 2023). Si et al. (2023) aimed at the issues of significant positioning and mapping errors caused by LiDAR point cloud distortion, designed a point cloud distortion correction method based on continuous-time trajectories of IMUs, established a data fusion model between LiDAR and IMUs, and proposed a positioning and mapping method for anti-collision drilling robots based on tightly coupled IMU-LiDAR, effectively improving the precision and performance of positioning and map building. Shen et al. (2023) addressed the problems of easy loss of GNSS signals and poor robustness of traditional SLAM algorithms in orchard environments by proposing a tightly coupled LiDAR/IMU framework. This framework optimizes IMU and LiDAR separately through a factor graph, enabling the IMU to output high-frequency pose information and integrating the LiDAR to construct accurate point cloud maps. Ye et al. (2019) introduced a tightly coupled LiDAR-IMU fusion method, which proposed a refined algorithm with rotation constraints to further align the LiDAR pose with the global map. All the above research utilizes the attitude information provided by IMU to offer a prior estimation for point cloud matching and distortion elimination.

Wheel speed sensor utilize dead reckoning to estimate the changes in a robot’s pose over time, assisting in robot positioning and capable of providing high-precision positioning accuracy in the short term (Wu et al., 2017). Ji et al. (2018) integrated LiDAR and wheel speed sensor information, using the Gmapping algorithm to establish an environmental map, thereby achieving positioning and navigation for inspection robots. Lu et al. (2024) received data from LiDAR and wheel speed sensor during the positioning and mapping process to determine the pose of an indoor disinfection robot and to create a two-dimensional grid map of the environment it occupies. Wang and An (2024) designed an autonomous navigation system for indoor medical goods transport and epidemic prevention assistant robots, utilizing the fusion of LiDAR and wheel speed sensor to complete positioning and mapping. High-precision wheel speed sensor can improve the point cloud distortion of LiDAR and enhance the precision of positioning and mapping in navigation.

Although the fusion of LiDAR with a single attitude transducer has improved the accuracy of localization and mapping to a certain extent, for the closed and narrow greenhouse working environments, and the uneven soil roads where electric crawler tractor operate, the precision and robustness of autonomous navigation are relatively weak. By adopting a method that combines LiDAR sensor with position-posture sensor, it is possible to obtain information about the characteristics of the greenhouse environment, information about obstacles surrounding the tractor, as well as the tractor’s own state information, improving the navigation accuracy and reliability of the electric tractor’s operation. Therefore, this study designs an autonomous navigation system for greenhouse electric track tractors based on LiDAR, integrating IMU and wheel speed sensor through an Extended Kalman Filter fusion, using the fused data to eliminate distortions in the LiDAR point cloud, thus enhancing the precision of localization and navigation.

## 3 Materials and methods

### 3.1 Design of the electric crawler tractor navigation system

#### 3.1.1 Agronomic parameter

The vegetables grown in the greenhouse can be categorized into leaf vegetables and fruit vegetables. The measurement of vegetable planting agronomic parameters is the key to the operation of electric crawler tractors. Generally, the height of vegetables in the harvest period falls within 50 cm, and the width of the greenhouse ranges between 6 to 16 m, which provides a reference for selecting the navigation hardware and determining the height of the sensor installation. According to the agronomic requirements, the autonomous navigation system of the electric crawler tractor is studied.

#### 3.1.2 Overall system composition

**Figure 2:**
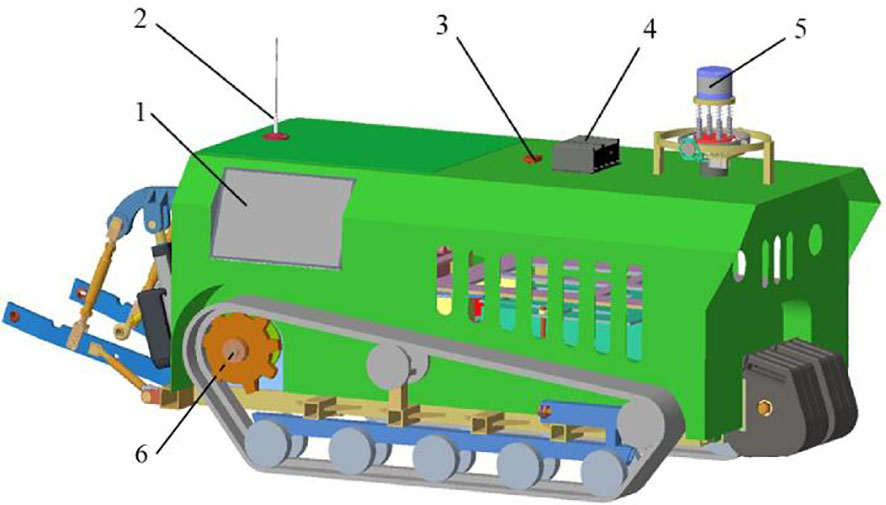
3D schematic diagram. 1. Vehicle display; 2. Antenna; 3. IMU; 4. Industrial computer; 5. LiDAR; 6. Wheel speed sensor.

In this paper, the navigation system is designed based on the remote-controlled electric crawler tractor, and the hardware of the navigation system is built, including the vehicle display, antenna, industrial computer, LiDAR,IMU, and wheel speed sensor. The navigation system enables the monitoring, environmental mapping, and releasing of navigation target point instructions through a remote monitoring platform on the PC and the vehicle display. The tractor provides real-time location information and working status feedback to both the PC remote monitoring platform and the vehicle display. The laser radar is installed on the fixed device at the front of the tractor; it obtains the environmental information of the greenhouse and feeds it back to the industrial computer to complete environmental information mapping. IMU is installed in the central position above the tractor; it measures the posture and acceleration of the tractor movement, assists the LiDAR in positioning, and sends the positioning information to the industrial computer, which sends the speed control information to the motor control system through CAN communication. Meanwhile, the motor controller feedbacks the real-time speed information of the chassis to the industrial computer through CAN communication. The tractor autonomous navigation task is completed by these components together, and the overall structure of the navigation system is illustrated in **Figure 2**.

#### 3.1.3 Hardware system design

**Figure 3:**
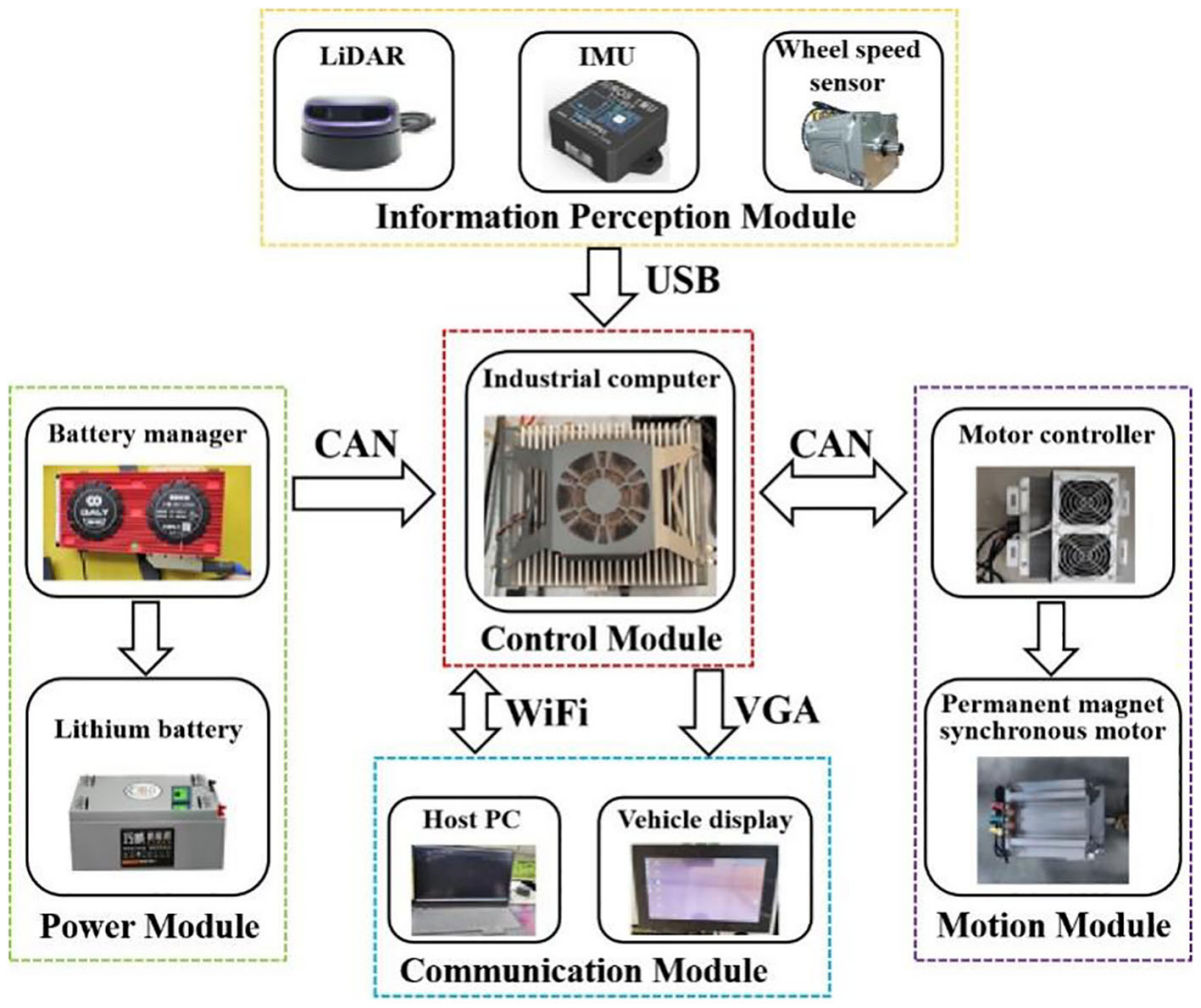
Overall hardware structure diagram.

The navigation hardware system of the electric crawler tractor mainly consists of five modules: the information perception module, the control module, the communication module, the motion module, and the power module. The structure of the hardware system is shown in **Figure 3**.

##### 3.1.3.1 Information perception module

The information perception module mainly includes LiDAR, IMU and wheel speed sensor. By using the RPLIDAR A2 2D LiDAR of Silan Technology and laser triangulation ranging technology, the laser triangulation measurement technology can perform up to 8,000 ranging actions per second, and the measuring distance can reach 25 m, which can meet the needs of mapping and navigation in the greenhouse environment. The IMU adopts a HIFI 6-axis inertial sensor produced by TAObotics company, whose return frequency can reach 300 Hz. It can obtain real-time and accurate attitude information of electric track tractors and coordinate with LiDAR for positioning in the greenhouse environment.

##### 3.1.3.2 Control module

The tractor takes an industrial computer as the top-level controller. The computer is equipped with an i3-9100T processor, 8 GB memory, and 128 GB hard disk, and it runs Ubuntu 16.04 and ROS Kinetic operating system. LiDAR and IMU are connected through the USB interface to achieve real-time communication, and real-time communication with two motor controllers and the battery management system is via CAN bus.

##### 3.1.3.3 Communication module

The industrial computer is connected to the remote monitoring platform of the host PC through a WiFi network, and it is connected to the vehicle display through VGA. The host PC is Lenovo Rescuer R9000P equipped with an R7-5800H processor and 16 GB memory. The vehicle display uses a 15-inch capacitive display to provide various interfaces. The remote monitoring platform on the PC and the vehicle display on the tractor can realize the monitoring of the tractor’s working status, environmental mapping, and the issuance of navigation target point instructions; the tractor, as the executor of the command, feedback real-time position information and working status to the host PC remote monitoring platform through the WiFi network and to the vehicle display through VGA. The industrial computer sends the speed control information to the motor controller through CAN communication, and the motor controller feeds back real-time chassis speed information to the industrial computer through CAN communication.

##### 3.1.3.4 Motion module

The tractor adopts the crawler-type differential drive motion mode, and the left and right driving wheels are controlled by an independent permanent magnet synchronous motor. When the tractor is working, the motor controller makes the tractor move by controlling the rotational speed of the permanent magnet synchronous motor, to realize the efficient and stable running of the tractor. The motor model is TZQ180-4-96B-X.

##### 3.1.3.5 Power module

In the power module, two pieces of 96 V lithium iron phosphate batteries are used to provide power for the tractor. Specifically, the charging power supply voltage is 220 V, the total capacity is 300 Ah, the rated operating power is 8 kW, and the power duration 9 h for transportation operations, which can meet the working time requirements in the greenhouse environment.

#### 3.1.4 Software system design

The navigation software system of the electric crawler tractor consists of three layers: the application layer, the data processing layer, and the execution layer. To be specific, the application layer is a navigation task scheduler based on Ubuntu, and the navigation system reaches any target point in the greenhouse environment according to user instructions. The data processing layer is a ROS-based mapping, positioning, navigation, and obstacle avoidance program. It is the core of the navigation software system and the key to realizing the autonomous navigation of electric crawler tractors. The execution layer is a tractor control program based on the Ubuntu open-source real-time operating system. It converts the speed information from the data processing layer to the underlying permanent magnet synchronous motor into the motor speed information, to realize the autonomous navigation of electric crawler tractors in the greenhouse environment (Hu, 2019).

### 3.2 Principle of the autonomous navigation function

The autonomous navigation process of electric crawler tractors mainly involves greenhouse environment map building, pose estimation of electric crawler tractors, global path planning, and local path planning.

#### 3.2.1 Greenhouse environment map construction

**Figure 4:**
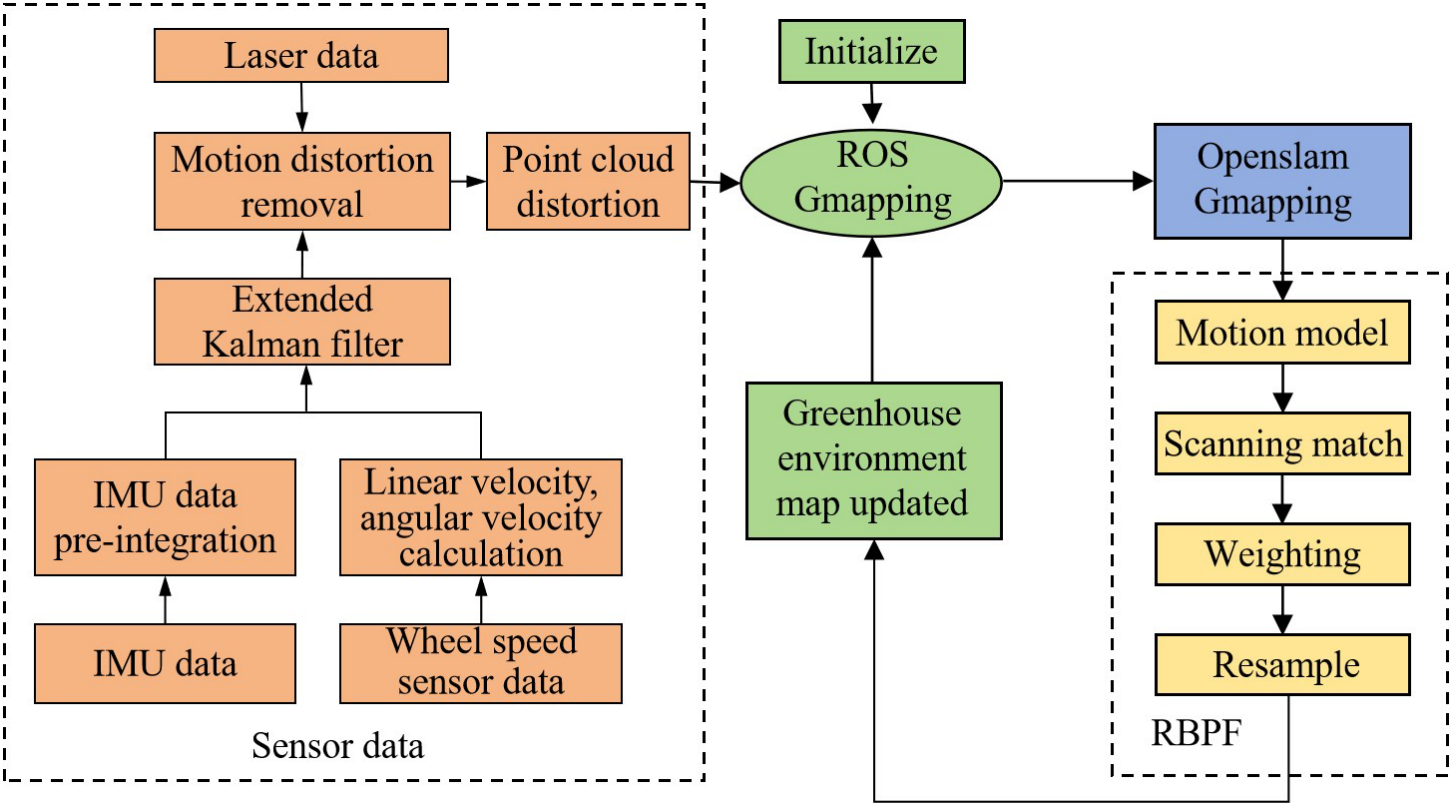
Gmapping algorithm-based flowchart for building electric crawler tractor.

In the design of autonomous navigation system for electric crawler tractor, greenhouse environment map is constructed based on Gmapping algorithm. Firstly, the environmental information is sensed by LiDAR, secondly, the extended Kalman filter algorithm is used to fuse the wheel speed sensor and IMU information to get the approximate position data of the tractor, and this data is used to remove the LiDAR point cloud distortion to get the position and control data of the tractor for a certain period of time to project the approximate trajectory of the tractor, and then the environmental map is projected according to this trajectory to build the corresponding environmental map finally. The flowchart of map building for motorized crawler tractor based on Gmapping algorithm is shown in **Figure 4**.

Gmapping algorithm is based on the particle filter algorithm (Rao-Blackwell zed Particle Filters, RBPF) and the 2D laser SLAM algorithm. The RBPF algorithm measures the complexity of the algorithm according to the number of particles required to build an environmental map. First, the approximate trajectory of the tractor is calculated using the pose and control data of the tractor in a certain period. Then, the environmental map is calculated according to this trajectory, and the corresponding environmental map is established (Mahmud, 2019). A posteriori estimate of the joint probability of a tractor is represented as Equations (1, 2):


(1) $$p(x_{1:t},m|z_{1:t},u_{1:t-1})$$

(2) $$p(x_{1:t},m|z_{1:t},u_{1:t-1})=p(m|x_{1:t},z_{1:t})p(x_{1:t}|z_{1:t},u_{1:t-1})$$

where *z*_1:_*_t_* denotes the data information observed by LiDAR in period 1 to *t*, *u*_1:_*_t_*_-1_ denotes the data controlled by the wheel speed sensor in period 1 to *t*-1, *x*_1:_*_t_* denotes the position and posture of the tractor at time *t*, *m* represents the raster map, *p*(*m*|*x*_1:_*_t_*,*z*_1:_*_t_*) represents the post-probability distribution estimates in the map, and *p*(*x*_1:_*_t_* |*z*_1:_*_t_*,*u*_1:_*_t_*_-1_) represents the posterior probability estimation of tractors.

Based on the RBPF algorithm, particle dissipation and computation are complex in pose estimation. The Gmapping algorithm proposes two improved methods: improved proposal distribution and selective resampling.

(1) Improving proposal distribution. By combining the motion information of the wheel speed sensor with the observation information of the LiDAR to obtain the next generation of particles more accurately, the proposed distribution is improved and gets closer to the target distribution. With an improved proposal distribution, the number of particles and the amount of algorithmic computation can be significantly reduced. The formula for particle weight update is given in Equations (3–7)


(3) $$w_{t}^{\left(i\right)}=w_{t-1}^{\left(i\right)}\frac{np(z_{t}|m_{t-1}^{\left(i\right)},x^{\left(i\right)})p(x_{t}^{\left(i\right)}|x_{t-1}^{\left(i\right)},u_{t-1})}{p(x_{t}^{\left(i\right)}|x_{t-1}^{\left(i\right)},u_{t-1})}\propto w_{t-1}^{\left(i\right)}\cdot p(z_{t}|m_{t-1}^{\left(i\right)},x_{t}^{\left(i\right)})$$

(4) $$p(x_{t}|m_{t-1}^{\left(i\right)},x_{t-1}^{\left(i\right)},z_{t},u_{t-1})=\frac{p(z_{t}|m_{t-1}^{\left(i\right)},x_{t})p(x_{t}|x_{t-1}^{\left(i\right)},u_{t-1})}{p(z_{t}|m_{t-1}^{\left(i\right)},x_{t-1}^{\left(i\right)},u_{t-1})}$$

(5) $$w_{t}^{\left(i\right)}=w_{t-1}^{\left(i\right)}\int^{}p(z_{t}|x')p(x'|x_{t-1}^{\left(i\right)},u_{t-1})dx$$

(6) $$u_{i}^{\left(t\right)}=\frac{1}{\eta^{\left(i\right)}}\sum_{i=1}^{k}p(z_{t}|m_{t-1}^{\left(i\right)},x_{j})p(x_{j}|x_{t-1}^{\left(i\right)},u_{t-1})\cdot \left(x_{j}-u_{t}^{\left(i\right)}\right){\left(x_{j}-u_{t}^{\left(i\right)}\right)}^{T}$$

(7) $$w_{t}^{\left(i\right)}=w_{t-1}^{\left(i\right)}p(z_{t}|m_{t-1}^{\left(i\right)},x_{t-1}^{\left(i\right)},u_{t-1})=w_{t-1}^{\left(i\right)}\cdot \eta^{\left(i\right)}$$

where *w*^(^*^i^*^)^*_t_* denotes the particle weight, *z_t_* denotes the most recent observation, *K* represents the number of simulated values, and *η* is the normalized factor.

(2) Selective resampling. In the Gmapping algorithm, selective resampling is used to determine whether to resample particles by setting an appropriate threshold and according to the dispersion of all particles’ weights (i.e., weight variance). Resampling is performed only when the weight of the particle is larger than the set threshold, thus reducing the number of resampling operations, slowing down the rate of particle degradation, and enhancing the accuracy of the algorithm.

#### 3.2.2 Pose estimation of the electric crawler tractor

**Figure 5:**
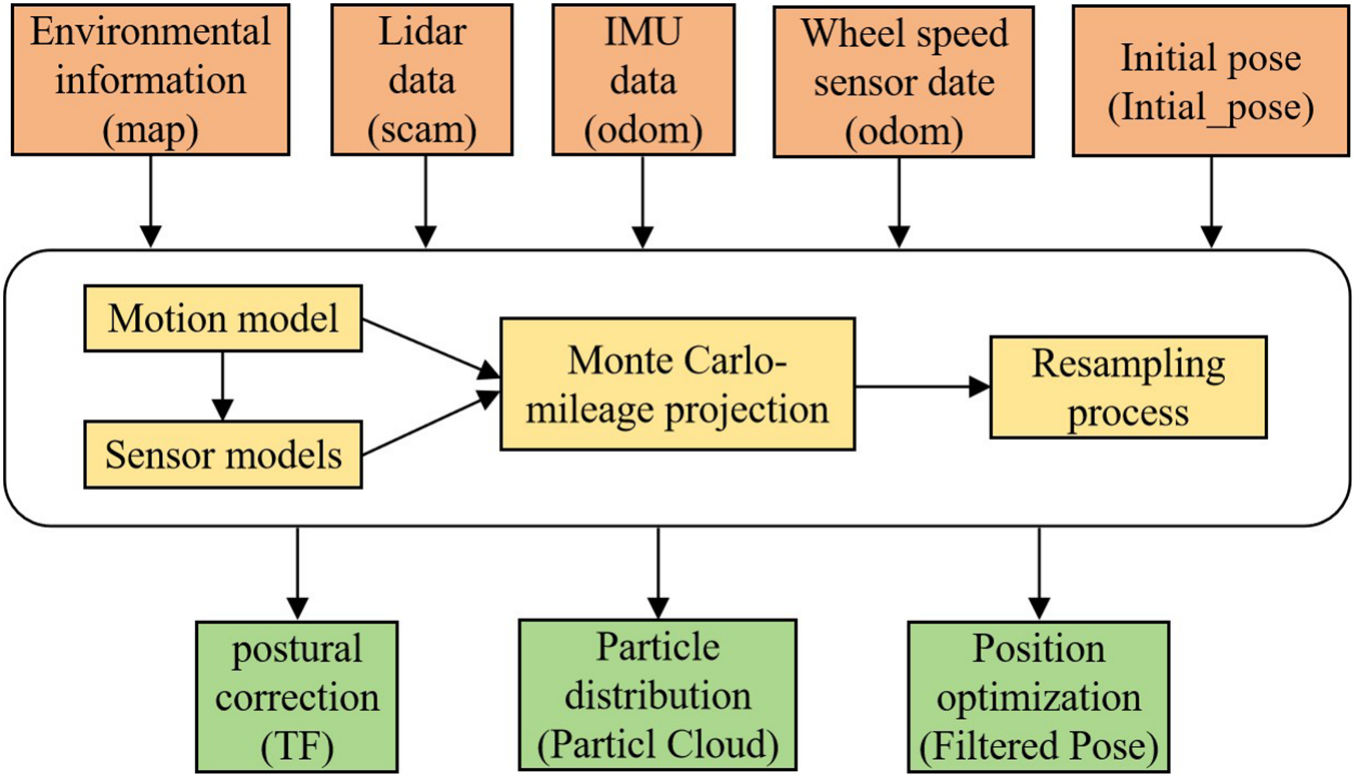
AMCL algorithm based electric crawler tractor positional positioning structure diagram.

The position of the electric crawler tractor in the current known greenhouse environment map is determined using the Adaptive Monte Carlo Localization (AMCL) algorithm. As a probabilistic statistical positioning method based on a particle filter, the AMCL algorithm uses the custom KLD method to update the number of particles, fuses the data of IMU and wheel speed sensor with the Kalman filter to maintain the convergence degree of particles, and estimates the probability distribution of tractor pose according to the sampled particles. The pose positioning of the electric track tractor based on the AMCL algorithm is demonstrated in **Figure 5**. Sufficient random particles are put into the whole pose space, and according to the LiDAR information, each particle is assigned a weight, and the weight of the sampled particles is updated. The larger the weight, the closer the pose is to the real pose.

When the electric crawler tractor is affected by external forces and deviates from the original path, the so-called “kidnap” phenomenon will lead to positioning failure. As the observed information changes, the weight of the particles will decrease, and the AMCL algorithm determines the change in the number of particles and the time by calculating the average weight of the short-term and long-term observation information, as in Equations (8, 9).


(8) $${\text{w}}_{\text{t}}={\text{w}}_{\text{l}}+{\text{a}}_{\text{l}}\left({\text{w}}_{\text{a}}-{\text{w}}_{\text{l}}\right)$$

(9) $${\text{w}}_{\text{s}}={\text{w}}_{\text{s}}+{\text{a}}_{\text{s}}\left({\text{w}}_{\text{a}}-{\text{w}}_{\text{s}}\right)$$

where *w_t_* and *w_s_* are the long-term and short-term average weights of the observation information, respectively; *a_l_* and *a_s_* are the long-term and short-term average weight coefficients of the observation information, respectively; *w_a_* is the average weight of the particles.

The probability of increasing the random particle population is as in Equation (10):


(10) $$\text{max}\left\{0.0,1.0-\frac{{\text{w}}_{\text{s}}}{{\text{w}}_{\text{l}}}\right\}$$

If *w_s_*/*w_l_* > 1, the algorithm determines that no random particles are added, and if *w_s_*/*w_l_* < 1, the algorithm will increase the number of particles in proportion to the two. Therefore, when the tractor “kidnap” phenomenon occurs, the short-term average weight of the observation information will suddenly decrease, and the AMCL algorithm will increase the number of particles to realize the adaptive repair of global positioning. The number of added particles has a great impact on the algorithm, and the number of added particles can be approximately determined by observing the measurement probability distribution of the sensor and combining the weight of the particles, as in Equation (11):


(11) $$\frac{1}{\text{M}}\sum_{\text{m}=1}^{\text{M}}{\text{w}}_{t}^{\text{m}}\approx \text{p}({\text{z}}_{\text{t}}|{\text{z}}_{1:\text{t}-1},{\text{u}}_{1:\text{t}},\text{m})$$

#### 3.2.3 Global path planning

**Figure 6:**
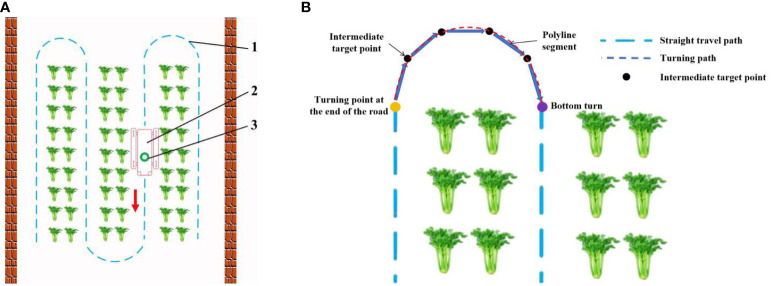
Schematic diagram of global path gauge for greenhouse operation of motorized crawler tractor. **(A)** Navigation path diagram. 1 represents a driving track; 2 represents motorized crawler tractor; 3 represents a LiDAR sensor. **(B)** The ground turning path planning diagram.

In the greenhouse environment, the driving path of the electric tractor includes straight-line driving and headland turning. To prevent the tractor from crushing crops in the headland turning process, this study adopts the multi-target point calibration method to perform global path planning, starting from the starting point of the headland turning, passing through several given intermediate target points, and finally reaching the endpoint of the headland turning. As illustrated in **Figure 6**, the local turning path is divided into several straight lines. Therefore, it is necessary to choose an algorithm with a short running time, fewer inflection points, and a short path length as the global path planning algorithm.

Common global path-planning algorithms include the Dijkstra algorithm, RRT algorithm (Rapid-exploration Random Trees), and A* algorithm (Guruji et al., 2016; Matoui et al., 2017).

**Figure 7:**
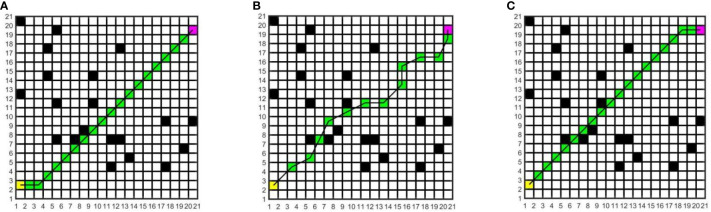
Simulation results of path planning for different algorithms in map 1. **(A)** Dijkstra algorithm. **(B)** RRT algorithm. **(C)** A* algorithm.

**Figure 8:**
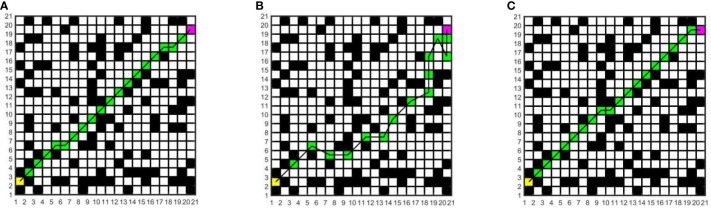
Simulation results of path planning for different algorithms in map 2. **(A)** Dijkstra algorithm. **(B)** RRT algorithm. **(C)** A* algorithm.

**Figure 9:**
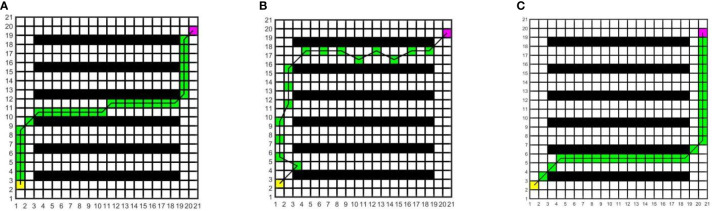
Simulation results of path planning for different algorithms in map 3. **(A)** Dijkstra algorithm. **(B)** RRT algorithm. **(C)** A* algorithm.

**Figure 10:**
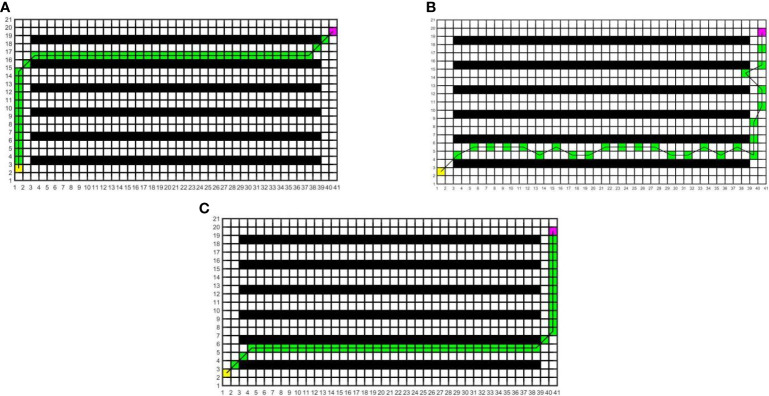
Simulation results of path planning for different algorithms in map 4. **(A)** Dijkstra algorithm. **(B)** RRT algorithm. **(C)** A* algorithm.

**Figure 11:**
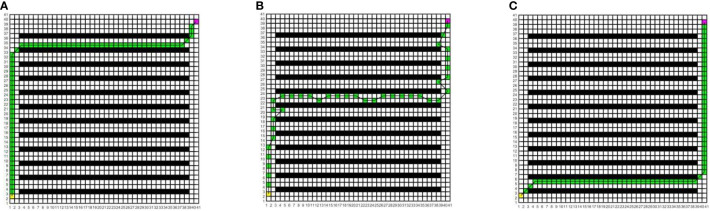
Simulation results of path planning for different algorithms in map 5. **(A)** Dijkstra algorithm. **(B)** RRT algorithm. **(C)** A* algorithm.

In this study, to determine the most suitable global path planning algorithm for the greenhouse planting environment, three different algorithms are compared and simulated in Matlab, and five different raster map scenes are constructed. Specifically, maps 1 to 3 have the same specifications but varying environmental complexity, and the size is 20×20; Maps 3 to 5 have the same environmental complexity but different specifications, and the sizes of maps 4 and 5 are 20×40 and 40×40, respectively. The Dijkstra algorithm, RRT algorithm, and A* algorithm are compared in five different raster maps, the same starting coordinates are set as (1.5, 2.5), and the end coordinates are set as (cols+0.5, rows-0.5), where cols is the set as the total number of columns in the raster map, and rows is the total number of rows. The yellow block represents the starting point, the purple block represents the endpoint, the white block represents the movable space, the black block represents the obstacle, the green block represents the path node, and the black solid line indicates the generated global path. The running environment of the algorithm is the 64-bit Windows 11 operating system, the PC is Lenovo Rescuer R9000P equipped with the R7-5800H processor and 16 GB memory, and the Matlab version is 2016a. The simulation results are shown in **Figures 7**–**11**.

In the five maps, map 1 is a simple environment with relatively few random arrangements of obstacles, map 2 is a chaotic environment with random arrangements of multiple obstacles, and maps 3 to 5 is a simulated greenhouse environment with relatively regular obstacles. For each raster map scene, three path planning algorithms are run 10 times. The initial parameter settings are consistent, and the evaluation indexes include the running time, number of inflection points, and path length. For each algorithm and each map, the average value of each evaluation index for 10 groups is taken, as shown in **Table 1**.

**Table 1:** Comparison of the running time, inflection points, and path length of different path planning algorithms.

Map scene	Path planning algorithm	Running time/s	Number of inflection points/size	Path length/cm
Map1	Dijkstra algorithm	0.062338	1	26.0416
	RRT algorithm	0.037272	10	28.3093
	A* algorithm	0.041013	1	26.0416
Map2	Dijkstra algorithm	0.060928	4	26.0416
	RRT algorithm	0.085479	11	33.4940
	A* algorithm	0.040065	3	26.0416
Map3	Dijkstra algorithm	0.061533	7	33.0711
	RRT algorithm	0.068319	12	37.8231
	A* algorithm	0.040961	3	33.0711
Map4	Dijkstra algorithm	0.096184	3	53.0711
	RRT algorithm	0.151257	15	57.7376
	A* algorithm	0.042955	3	53.7011
Map5	Dijkstra algorithm	0.168815	5	73.7011
	RRT algorithm	0.283979	24	79.4787
	A* algorithm	0.052560	3	73.0711

As can be seen from **Table 1**, in the five map scenes, the path length planned by the A* algorithm and Dijkstra algorithm is the same, which is reduced by 8.01%, 22.25%, 12.56%, 8.08% and 7.27% respectively compared with the RRT algorithm. In the scenarios of map 1 and map 4, the A* algorithm has the same number of inflection points as the Dijkstra algorithm, which is reduced by 90.00% and 80.00%respectively compared with the RRT algorithm. In the scenes of map 2, map 3, and map 5, the number of inflection points of the A* algorithm is reduced by 25.00% and 63.64%, 57.14% and 75.00%, 40.00% and 87.50% respectively compared with other algorithms. In the scenario of map 1, the running time of the A* algorithm is less than that of the Dijkstra algorithm and more than that of the RRT algorithm. In the scenes of maps 2 to 5, the running time of the A* algorithm is reduced by 34.24% and 53.13%, 33.43% and 40.04%, 55.34% and 71.60%, 68.87% and 81.49% respectively, compared with other algorithms. With the increase in obstacle complexity and map size, the RRT algorithm generates more path turning points, and its running time increases significantly. Compared with the RRT algorithm, the Dijkstra algorithm obtains a shorter path and fewer turning points, but it is more time-consuming than the A* algorithm, and the running time increases greatly with the map scale. The A* algorithm obtains the best global path, the path length and running time are shorter, and the improvement effect of search efficiency is more obvious with the increase in the complexity of environment maps. Among them, in map 5, the path planned by the A* algorithm is significantly superior to those of other algorithms. Therefore, this paper adopts the A* algorithm for global path planning of electric crawler tractors in the greenhouse environment.

The A* algorithm provides an efficient direct search method for obtaining the shortest path in the static road network. By combining the advantages of the Dijkstra algorithm and the BFS (Breath First Search) algorithm, it can find an optimal path based on the cost function while improving the efficiency of the algorithm through heuristic search. The cost function of the A* algorithm is given in Equation (12):


(12) f(n)=g(n)+h(n)

where *f*(*n*) is the cost estimate from the initial state through state n to the target state, *g*(*n*) is the actual cost of going from the initial state to state n in the state space, *h*(*n*) is the estimated cost of the best path from state n to the destination state.

The A* algorithm searches in the direction of approaching the target, and it inspects every node in the search direction during the search process. When a node is reached, the surrounding nodes of the node will be added to OpenList. The node with the smallest estimated value in OpenList will be selected as the next expansion node and added to ClosedList. The process will be repeated until the target node is added to OpenList. When a global planning trajectory from the target point back to the starting point is formed, the pathfinding process is considered successful.

#### 3.2.4 Local path planning

In actual situations, there may be unknown obstacles on the original navigation path of electric crawler tractors. Considering this, based on global path planning, this paper adopts the DWA algorithm to detect local environmental information through LiDAR and achieve real-time obstacle avoidance.

##### 3.2.4.1 Kinematics model

**Figure 12:**
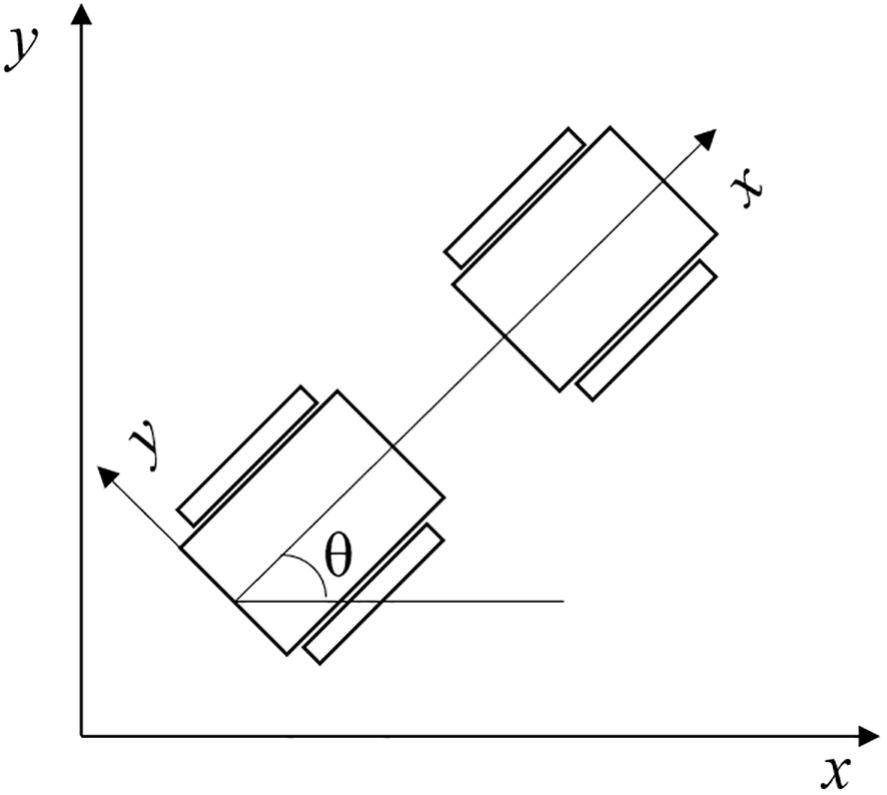
Differential kinematics model for electric tracked tractor.

The DWA algorithm converts the position control of the tractor into speed control. To use speed sampling to predict the motion trajectory of the electric crawler tractor, the motion model of the electric crawler tractor needs to be analyzed first. The differential kinematic model of the electric crawler tractor is shown in **Figure 12**.

The DWA algorithm uses the tractor motion model for trajectory simulation and finds the best path in several simulated trajectories. Let *v*(*t*) and *ω*(*t*) represent the linear and angular velocity of the tractor at time *t* in the world coordinate system, respectively (Mahmud et al., 2019; Molinos et al., 2019). In the sampling period *t*, the displacement is small, and the tractor moves in a straight line at an approximately uniform speed. Then, the pose increment of the two adjacent moments is as in Equation (13):


(13) $$\{\begin{array}{l} \Delta x=v_{t}\Delta tcos\left(\theta_{t}\right)\\ \Delta y=v_{t}\Delta tsin\left(\theta_{t}\right)\\ \Delta \theta =\omega \Delta t \end{array}$$

The pose at time *t*+1 can be expressed as in Equation (14):


(14) $$\{\begin{array}{l} x\left(t+1\right)=x\left(t\right)+v_{t}\Delta tcos\left(\theta_{t}\right)\\ y\left(t+1\right)=y\left(t\right)+v_{t}\Delta tsin\left(\theta_{t}\right)\\ \theta \left(t+1\right)=\theta \left(t\right)+\omega \Delta t \end{array}$$

where *x*(*t*), *y*(*t*), *θ*(*t*) – the position and posture of the tractor in the world coordinate system at time *t*.

##### 3.2.4.2 Velocity sampling

In the actual greenhouse environment, the DWA algorithm transforms the obstacle avoidance problem into three-speed constraints of the electric crawler tractor in the speed space, including the maximum and minimum speed constraints of the tractor, the motor acceleration and deceleration constraints, and the braking distance constraints. The above constraints can restrict the movement speed of the tractor within a certain range, and the specific constraints are represented as follows:

###### 3.2.4.2.1 Maximum and minimum speed constraints

Since the hardware performance of the tractor sets limitations on the minimum and maximum speeds of the tractor, the sampling speed of the tractor should be controlled within the interval of the optimal angular speed and linear speed of the tractor, and the constraint is given in Equation (15)


(15) $$V_{m}=\left\{\left(v,\omega \right)|v\in \left[v_{min},v_{max}\right],\omega \in \left[\omega_{min},\omega_{max}\right]\right\}$$

where *v_max_* and *v_min_* are tractor maximum line speed and minimum line speed, and *ω_min_* and *ω_max_* are maximum angular speed and minimum angular speed of the tractor.

###### 3.2.4.2.2 Motor acceleration and deceleration constraints

The acceleration of the tractor is subject to the output torque of the motor, and the space sampling of the velocity vector should fall within the tolerable range of the motor torque. The constraint is represented as in Equation (16)


(16) $$V_{d}=\left\{\left(v,\omega \right)|v\in \left[v_{c}-{\dot{v}}_{b}\Delta t,v_{c}+{\dot{v}}_{a}\Delta t\right],\omega \in \left[\omega_{c}-{\dot{\omega }}_{b}\Delta t,\omega_{c}+{\dot{\omega }}_{a}\Delta t\right]\right\}$$

where *v_c_* is current linear velocity, *ω_c_* is the current angular velocity, ${\dot{v}}_{a}$ is maximum linear acceleration, ${\dot{\omega }}_{a}$ is maximum angular acceleration, ${\dot{v}}_{b}$ is maximum line deceleration, ${\dot{\omega }}_{b}$ is maximum angular deceleration.

###### 3.2.4.2.3 Braking distance constraints

To guarantee that the tractor stops before hitting a random obstacle, the linear speed and angular speed of the tractor before reaching the obstacle should be reduced to 0 under the condition of maximum deceleration. The constraint is expressed as in Equation (17)


(17) $$V_{a}=\left\{\left(v,\omega \right),v\leq \sqrt{2dist\left(v,\omega \right){\dot{v}}_{b}},\omega \leq \sqrt{2dist\left(v,w\right){\dot{\omega }}_{b}}\right\}$$

where *dist*(*v, ω*) is the nearest distance between the tractor and the obstacle.

Finally, the speed of the electric crawler tractor needs to take the intersection of the above three constraint spaces, i.e., the dynamic window speed *V_r_* should satisfy the following condition, as shown in Equation (18):


(18) $$V_{r}=V_{m}{⋂}^{\;}V_{d}{⋂}^{\;}V_{a}$$

###### 3.2.4.2.4 Evaluation function

The DWA algorithm evaluates the simulated trajectories in the set of speed groups, selects the motion trajectories with the best performance and sends them to the chassis of the tractor, controls the tractor to finish the evading task, and evaluates the trajectories using the evaluation function. The evaluation function is as in Equation (19)


(19) G(v,ω)=σ(α·heading(v,ω)+β·dist(v,ω)+γ·velocity(v,ω))

The evaluation functions of the DWA algorithm involve azimuth angle, linear velocity, and the nearest distance between simulated trajectory and obstacles, and each evaluation function needs to be normalized (Zhang, 2018; Mahmud et al., 2019), as shown in Equations (20–22):


(20) $$normal\_heading\left(i\right)=\frac{heading\left(i\right)}{\sum_{i=1}^{n}heading\left(i\right)}$$

(21) $$normal\_dist\left(i\right)=\frac{dist\left(i\right)}{\sum_{i=1}^{n}dist\left(i\right)}$$

(22) $$normal\_velocity\left(i\right)=\frac{velocity\left(i\right)}{\sum_{i=1}^{n}velocity\left(i\right)}$$

where *heading *(*v, ω*) is the azimuth evaluation subfunction evaluates the azimuth deviation between the direction of the end of the simulated trajectory and the target at the current speed; *dist* (*v*, *ω*) is the obstacle evaluation subfunction represents the closest distance between the simulated trajectory of the corresponding velocity group and the obstacle. If there is no obstacle on the current trajectory, *dist *(*v*, *ω*) is set as a constant; *velocity* (*v*, *ω*) is the velocity evaluation subfunction represents the velocity magnitude of the simulated trajectory; *σ* is smoothing function; *α* is direction influence coefficient, the closer the tractor is to the target point, the greater the *α* value; *β* is safety distance factor, the closer the tractor is to the obstacle, the greater the *β* value; *γ* is velocity influence coefficient, the higher the speed of the tractor, the greater the gamma value.

## 4 Results and discussion

**Figure 13:**
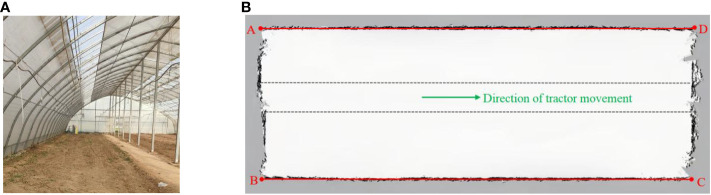
Greenhouse environments and maps created by the Gmapping algorithm. **(A)** Greenhouse environment. **(B)** Map created by Gmapping algorithm.

The demonstration greenhouse of the Rougu fruit and vegetable professional cooperative in Yangling District of Shaanxi Province is selected to test the mapping performance and navigation accuracy respectively. The greenhouse environment is relatively closed, the size of the greenhouse is 43 m×15.5 m, and the middle road is a relatively flat cement road with regular distribution, and vegetable planting areas are on both sides of the road, as demonstrated in **Figure 13A**.

### 4.1 Construction accuracy test

Before the drawing test, the systematic error of the wheel speed sensor of the electric crawler tractor is corrected to reduce the positioning error. The drawing test of the electric crawler tractor was conducted on the transport channel of the greenhouse. The transport channel was relatively smooth, and the slippage between the track and the ground was small. The remote-controlled tractor moves slowly in the greenhouse and uses LiDAR to obtain information about the surrounding environment. However, the LiDAR sensor will shake when gullies are encountered. Therefore, the remote-controlled tractor needs to scan and build the map back and forth to ensure that the environmental map is complete.

The built environment map is shown in **Figure 13B**. In this figure, the solid red line is the reference line at both ends of the greenhouse, the green arrow indicates the direction of tractor movement, and the dotted black line denotes the cement road taken by the map construction. Four map endpoints A, B, C, and D are selected respectively, and the distance between the adjacent endpoints is compared with the actual size of the map construction area, as listed in **Table 2**.

**Table 2:** Relative error of facility environment map.

Position	Real value/m	Atlas value/m	Misalignment/m
AB	15.50	15.46	0.04
BC	43	43.16	0.16
CD	15.50	15.44	0.06
AD	43	42.87	0.13

It can be seen from **Table 2** that the tractor will continuously produce cumulative errors during mapping, leading to a slight distortion at the edge of the map. The maximum lateral deviation of the built environment map is 6 cm and the maximum longitudinal deviation is 16 cm, which can meet the map accuracy requirements for navigating electric crawler tractors in the greenhouse.

### 4.2 Navigation accuracy test

The speed of the electric crawler tractor is set to 1 m/s, the greenhouse environment map is loaded, and the position and posture of the starting point of the tractor operation and the target point and posture of the navigation are set. The AMCL algorithm is employed to obtain real-time position information of the electric crawler tractor, and the A* algorithm is utilized to plan the globally optimal path from the current position of the tractor to the navigation target point. The environment map after expansion is obtained by real-time detection of environmental obstacle information by LiDAR. When an obstacle is detected, the scanned laser point cloud information is fed into the DWA algorithm to replan the optimal path of the tractor.

The test was conducted in five groups with a test distance of 20 m. In each group of tests, data sampling was measured at an interval of 2 m. After the end of navigation, the lateral deviation and heading deviation of the tractor at each sampling point were measured. The left side of the target point is positive deviation, the right side of the target point is negative deviation, the actual course on the left side of the target course is positive deviation, and the actual course on the right side of the target course is negative deviation. The mean deviation and standard deviation of the tracking path were calculated based on the test data. After each test, the initial position of the tractor was reset to prevent the accumulation of errors from affecting the test results. The test results are presented in **Table 3**.

**Table 3:** Navigation accuracy test results.

Test serial number	Lateral deviation/cm			Heading deviation/^°^		
	Maximum	Average	Standard deviation	Maximum	Average	Standard deviation
1	-12.40	+6.78	7.90	-13.30	-6.52	7.16
2	-11.80	-6.34	7.42	-12.30	4.48	5.72
3	+8.80	-4.69	5.65	+11.20	5.85	6.56
4	11.60	+6.44	7.01	+10.40	-5.73	6.59
5	-9.50	-5.34	6.06	-12.20	-6.07	7.22

It can be seen from **Table 3** that after five tests in the greenhouse, the maximum transverse deviation is no more than 13cm, the average transverse deviation is less than 7cm, and the mean standard deviation is less than 8cm. The maximum heading deviation is less than, the average heading deviation is less than 14^°^, and the standard deviation is less than 7^°^. These results indicate that the system developed in this study can meet the accuracy requirements for autonomous navigation of the electric crawler tractor in the greenhouse.

## 5 Conclusion

To improve the intelligence level of electric crawler tractors in the greenhouse, based on LiDAR technology, this paper designs an autonomous navigation system of electric crawler tractors for the greenhouse planting environment. The hardware part mainly consists of LiDAR, IMU, wheel speed sensor, industrial computers, etc. The software core control layer is developed based on ROS, and information exchange is realized through distributed node communication. The Gmapping algorithm is employed to build the greenhouse environment map. The mapping test shows that the maximum lateral deviation of the built map is 6cm, and the maximum longitudinal deviation is 16cm, which can meet the map accuracy requirements in the greenhouse. To select the path planning algorithm suitable for greenhouse operations, common path planning algorithms are simulated, and the results suggest that the A* algorithm obtains the best global path, its path length and running time are shorter, and the improvement effect of search efficiency is more obvious with the increase in the complexity of the environment map. Therefore, the A* algorithm is used in this study for global path planning, and the DWA algorithm is used for local path planning. The accuracy test results of the navigation system indicate that the maximum lateral deviation is less than 13cm, the average lateral deviation is less than 7cm, and the standard deviation is less than 8cm. Meanwhile, the maximum heading deviation is no more than, the average heading deviation is less than 7^°^, and the standard deviation is less than 8^°^. The accuracy meets the navigation and positioning requirements of electric crawler tractors in greenhouse transportation. At present, this study can only solve the simple positioning and navigation problems in the greenhouse. In future work, different navigation strategies will be formulated according to the specific operation conditions of the greenhouse, and the positioning and navigation accuracy can be further enhanced.

## Data availability statement

The original contributions presented in the study are included in the article/supplementary material. Further inquiries can be directed to the corresponding author.

## Author contributions

HG: Funding acquisition, Project administration, Supervision, Writing – review & editing. YL: Investigation, Methodology, Writing – original draft. HW: Investigation, Methodology, Writing – original draft. TW: Data curation, Validation, Writing – original draft. LR: Data curation, Validation, Writing – original draft. HW: Validation, Writing – original draft. ZW: Validation, Writing – original draft. CW: Validation, Writing – original draft. JZ: Validation, Writing – original draft. YH: Visualization, Writing – review & editing. SG: Visualization, Writing – review & editing.
